# Short-interval intravenous indocyanine green administration in pediatric laparoscopic cholecystectomy: a prospective evaluation of visualization and safety

**DOI:** 10.1007/s00383-025-06172-x

**Published:** 2025-08-26

**Authors:** Vojtech Dotlacil, Eliska Pajerova, Dagmar Sovadinova, Barbora Kucerova, Martin Vyhnanek, Michal Rygl

**Affiliations:** Department of Paediatric Surgery, Second Faculty of Medicine, Charles University and Motol University Hospital, Charles University, V Uvalu 84, Praha 5, 150 06 Prague, Czech Republic

**Keywords:** Indocyanine green, Fluorescence imaging, Pediatric cholecystectomy, Biliary visualization, Short-interval ICG administration

## Abstract

**Purpose:**

Indocyanine green (ICG) fluorescence imaging enhances biliary visualization during pediatric laparoscopic cholecystectomy (LC), helping to identify anatomical variants and prevent bile duct injury. Standard pediatric recommendations suggest ICG administration 16–24 h preoperatively; however, this may be impractical. This study aims to evaluate the safety and effectiveness of short-interval ICG administration.

**Methods:**

A prospective single-center study (October 2024–June 2025) included pediatric LC patients receiving intravenous Verdye® preoperatively. Visualization of extrahepatic biliary anatomy was assessed intraoperatively using a 5-point Likert scale, HELPFUL (usefulness), and DISTURBED (liver background interference) scores. Data included indication, ICG timing, operative time, and complications according to the Clavien–Dindo classification (C–D).

**Results:**

Eleven patients (64% female), median age 14 years (IQR 12,7–15,7) and median weight 65,5 kg (IQR 46,5–80), were included. Five had BMI > 25 kg/m^2^; five (46%) underwent preoperative ERCP. ICG (median dose 0.34 mg/kg) was administered a median of 225 min before surgery. Median operative time was 65 min (IQR 58–68). Median Likert score was 5; HELPFUL 3; DISTURBED 1. No ICG-related or C–D complications occurred.

**Conclusion:**

Short-interval ICG administration was safe, feasible, and effective in enhancing biliary visualization during pediatric LC. This approach was well-tolerated and provided high-quality imaging without complications.

**Supplementary Information:**

The online version contains supplementary material available at 10.1007/s00383-025-06172-x.

## Introduction

Laparoscopic cholecystectomy (LC) has become the gold standard for treating symptomatic cholelithiasis in children. In addition to the typical symptoms of biliary colic, this condition can lead to a spectrum of serious complications, including acute cholecystitis, choledocholithiasis, acute cholangitis, biliary pancreatitis, and biliodigestive fistulas [1]. These complications contribute to significant morbidity and, in some cases, mortality.

However, anatomical variations and inflammatory changes increase the risk of bile duct injury during dissection. This risk may be further amplified in pediatric surgery, where the lower case volume compared to adults limits routine familiarity with the procedure. Conventional intraoperative cholangiography can assist in visualizing the biliary tree; however, it has several drawbacks—it typically requires bulky fluoroscopy equipment, exposes both patients and staff to ionizing radiation, and necessitates additional personnel. Moreover, cannulation of the bile duct for contrast injection carries a risk of iatrogenic injury and is time-consuming [2–5].

Near-infrared fluorescence cholangiography (NIFC) using intravenous indocyanine green (ICG) enhances intraoperative visualization of biliary structures and has been shown to improve surgical safety, particularly in settings where cholecystectomy is performed less frequently [6–12].

Recent pediatric studies recommend administering ICG 16–24 h [7, 13] prior to surgery to allow hepatic uptake and biliary excretion, thereby ensuring optimal fluorescence while minimizing interference from background liver fluorescence. However, such prolonged intervals pose practical limitations—including extended hospitalization, the need for additional intravenous access, and scheduling constraints. Moreover, shorter intervals between ICG administration and surgery may increase the likelihood that the planned procedure will proceed as scheduled, especially in elective settings where operating room availability is limited and more urgent cases may take precedence—potentially rendering the administered dye useless. In addition, the long delay between administration and surgery effectively precludes the use of ICG in urgent or acute cases, where the available preoperative interval is inherently short [14, 15].

Existing evidence, although limited, suggests that shorter preoperative intervals may still provide adequate biliary visualization in adults [16–18]; however, pediatric-specific data are lacking. To address this gap, we prospectively evaluated the feasibility, effectiveness, and safety of short-interval ICG administration prior to laparoscopic cholecystectomy in pediatric patients.

## Methods

### Study design and patient population

This prospective observational study was conducted at the Department of Paediatric Surgery, Motol University Hospital, Prague, Czech Republic, between October 1, 2024 and June 30, 2025. All pediatric patients under the age of 18 who were scheduled for laparoscopic cholecystectomy were screened for eligibility.

### Inclusion and exclusion criteria

Patients were eligible for inclusion if they were younger than 18 years, had an indication for laparoscopic cholecystectomy, and had provided informed consent from a parent or legal guardian, along with assent from the patient when appropriate. Exclusion criteria included known allergy to ICG or iodine-based contrast agents, severe hepatic dysfunction, emergency procedures in which ICG could not be administered preoperatively, and lack of signed informed consent.

### ICG administration and surgical technique

All enrolled patients received intravenous indocyanine green (Verdye®) at a dose of 0.30–0.5 mg/kg, diluted in aqua pro injection (1 mg = 5 ml). The dye was administered within a shorter interval prior to surgery.

Laparoscopic cholecystectomy was performed using a standard four-port technique. LC was performed following the criteria for safe LC [12, 19]. Intraoperative fluorescence imaging was performed using the Olympus VISERA ELITE III (Olympus Medical Systems Corp., Tokyo, Japan) imaging platform equipped with near-infrared fluorescence capabilities.

### Assessment of biliary fluorescence

The quality of biliary fluorescence was evaluated intraoperatively by the lead surgeon using a five-point Likert scale. A score of 5 indicated excellent visualization of biliary anatomy including the cystic duct and common bile duct; a score of 4 indicated good visualization with only mild limitations; a score of 3 indicated partially visible structures; a score of 2 indicated poor fluorescence; and a score of 1 indicated complete absence of visible fluorescence.

Additionally, two auxiliary parameters were recorded for each case:

HELPFUL score—reflecting the perceived utility of fluorescence imaging in the specific surgical context. This was graded on a 4-point scale:

0 = not helpful;

1 = moderately helpful (increased surgeon’s intraoperative confidence);

2 = very helpful (enabled safer dissection);

3 = highly helpful (potentially prevented biliary injury).

DISTURBED score—evaluating the extent to which background fluorescence from the liver interfered with biliary structure visualization. This was graded from 0 to 4:

0 = no disturbing liver fluorescence;

1 = minimally disturbing (all relevant structures clearly visible in NIR mode);

2 = mild disturbance, though cystic duct–common bile duct (CD-CBD) junction still visible prior to dissection;

3 = moderate disturbance, CD-CBD junction visible only after dissection;

4 = severe disturbance, anatomical orientation not feasible due to overwhelming background fluorescence [20].

### Clinical variables and outcomes

For each patient, the following data were collected: age, sex, body mass index (BMI), surgical indication and timing (categorized as elective, subacute, or acute), total interval between ICG administration and incision (in minutes), administered dose of ICG (Verdye) in milligrams, operative time (skin-to-skin), intraoperative findings including any anatomical variations, occurrence of intraoperative or postoperative complications, and the need for conversion to open surgery. Postoperative complications occurring within 30 days were recorded and graded according to the Clavien–Dindo classification (C–D) system. Any adverse events related to ICG administration were also documented. In addition, the lead surgeon provided a subjective rating of satisfaction with ICG fluorescence imaging.

The primary outcome of the study was the quality of biliary fluorescence based on the intraoperative Likert scale assessment. Secondary outcomes included operative time, frequency and severity of perioperative complications, and adverse reactions to ICG.

### Statistical analysis

Continuous variables are presented as medians with interquartile ranges (IQR), and categorical variables as counts and percentages. All data analyses were performed using standard spreadsheet and statistical software.

### Ethical approval

The study protocol was reviewed and approved by the institutional ethics committee of Motol University Hospital—Reference No.: EK160-25. All procedures were conducted in accordance with the Declaration of Helsinki.

## Results

A total of 11 pediatric patients underwent laparoscopic cholecystectomy with the use of ICG between October 1, 2024, and June 31, 2025. There were 7 (64%) girls. The median weight at the time of surgery was 65,5 kg (IQR 46,5–80), and five (46%) patients had a BMI > 25 kg/m^2^. Comorbidities were present in six (54,5%) patients (3 × hereditary spherocytosis, 1 × hirsutism, 1 × dysfibrinogenemia, 1 × dilated cardiomyopathy).

All patients underwent surgery for symptomatic cholelithiasis. Five (46%) patients underwent preoperative endoscopic retrograde cholangiopancreatography (ERCP), and all underwent cholecystectomy during the index hospitalization. The median time from ERCP to surgery was 3 days (IQR 1–4). The median age at the time of surgery was 14 years (IQR 13–16). None of the patients received antibiotic therapy.

All procedures were performed laparoscopically using a 4-port technique, and no conversion to laparotomy was required. No patient required intraoperative placement of a surgical drain. The median duration of surgery was 65 min (IQR 58–68). All patients received combined postoperative analgesia and were allowed oral fluids and a light meal on the same day as surgery. The median length of postoperative hospital stay was 2 days (IQR 2–3).

No adverse reactions related to ICG administration were observed. Within 30 days postoperatively, no C–D complications occurred. All patients attended the scheduled follow-up and no readmissions or reinterventions were necessary.

In all cases, ICG was administered intravenously preoperatively. The median Verdye dose was 0.34 mg/kg (IQR 0.31–0.36), administered at a median interval of 225 min (IQR 210–310) prior to surgery.

### Fluorescence visualization and surgeon satisfaction

Evaluation of fluorescence quality was recorded in all cases. The median HELPFUL score was 3 (IQR 2,5–3), indicating that surgeons generally found the use of ICG highly beneficial.

The DISTURBED score, measuring the degree of interference from liver background fluorescence, reached a median value of 1 (IQR 0–1,5). Most procedures were not negatively affected by background signals; however, in two cases the visualization of the CD–CBD junction was partially impaired and became clear only after dissection. No case reached the highest disturbance level (score 4).

Surgeon satisfaction with fluorescence imaging, as measured on a 5-point Likert scale, reached a median score of 5 (IQR 4,5–5). In nine of eleven cases, the surgeon either fully or strongly agreed with the usefulness of ICG.

No subjective increase in cognitive load was observed with the use of fluorescence compared to conventional laparoscopic cholecystectomy.

## Discussion

To our knowledge, this is the first prospective study in the pediatric population specifically designed to evaluate the efficacy and safety of short-interval intravenous administration of ICG for near-infrared fluorescence cholangiography during laparoscopic cholecystectomy. Our findings suggest that shorter preoperative intervals can provide sufficient biliary visualization, with high surgeon satisfaction and no observed perioperative complications or adverse events.

### Safety and tolerance of ICG

ICG is a water-soluble, non-toxic dye that, upon intravenous administration, rapidly binds to plasma proteins—primarily albumin—in the bloodstream. It is subsequently extracted by the liver without undergoing metabolism and is excreted exclusively into the bile [21]. Although allergic reactions to ICG—including urticaria, anaphylaxis, and cardiovascular collapse—have been reported in the literature, particularly in adults undergoing cardiopulmonary procedures [22], no adverse reactions were observed in our cohort or in previous pediatric studies using ICG fluorescence cholangiography [23–26].

Nevertheless, ICG administration should be avoided in patients with hepatic failure, iodine allergy, a history of allergic reaction to ICG, or thyroid dysfunction. Additionally, caution is warranted in neonates with hyperbilirubinemia, as ICG can displace bilirubin from albumin binding sites, increasing the level of unbound bilirubin and potentially leading to kernicterus—a serious and harmful condition in newborns.

### Optimal timing of ICG administration

Optimal timing and dosage of ICG are crucial for its effective application; however, a standardized protocol has not yet been established. In the adult population, there are discrepancies in administration timing. For example, the results of the DOSE clinical trial [18] suggest that, regardless of ICG dose, administration more than 3 h before surgery reduces liver background fluorescence in the NIFC during LC. In contrast, Kinami et al. reported [27] that the most effective timing for ICG administration—resulting in optimal reduction of liver background fluorescence and enhanced visualization of the extrahepatic bile ducts—was 20 h prior to surgery, using a dosage of 25 mg/BW. Similarly, in the pediatric population, Esposito et al. observed that administering ICG from 16 to 18 h preoperatively provided an optimal bile duct-to-liver fluorescence ratio. Based on these findings, they standardized the timing of administration to a median of 15 h preoperatively, with an optimal dosage of 0.35 mg/kg [23]. This approach, however, requires earlier hospital admission and certainty that the operation will proceed as scheduled the following day. In contrast, alternative approaches have been explored for emergency and subacute cases, where such prolonged preoperative intervals are impractical or unfeasible. In these scenarios, the ability to administer ICG shortly before surgery holds particular value. Some authors have even described direct intraoperative injection of ICG into the gallbladder to achieve immediate biliary visualization [28]. Supporting this strategy, Pavulans et al. [29] demonstrated that even in patients with acute mild-to-moderate cholecystitis, ICG administration shortly before to LC significantly enhanced visualization of the extrahepatic biliary ducts.

In addition to timing, other factors may influence the quality of biliary visualization. For instance, a higher BMI has been associated with reduced fluorescence quality [30], likely due to the limited penetration depth of near-infrared light, which typically reaches only 5–10 mm into tissue [31]. In our cohort, among four patients with a BMI above 25, visualization of the biliary tree was not achieved in one case.

### Surgeon satisfaction and visualization outcomes with ICG

These results indicate that even shorter preoperative intervals can provide sufficient biliary visualization, with high surgeon satisfaction and no observed perioperative complications or adverse events. The use of ICG fluorescence was rated as highly helpful in the majority of our cases, with surgeons reporting improved anatomical orientation and enhanced safety during the dissection of Calot’s triangle. These findings align with those of recent studies showing a reduced complication rate and shorter dissection time when ICG is used [16, 23, 32]. Notably, the visualization rate of the cystic duct and common bile duct in our study is consistent with results from other pediatric cohorts using longer preoperative intervals [13], and aligns with adult studies reporting > 90% duct identification after dissection [17, 29].

In our cohort, the LCs were performed electively or subacutely. The administration interval had a median of 225 min preoperatively, with a median Verdye dose of 0.34 mg/kg. Using this protocol, we achieved a median Likert score of 5, a median HELPFUL score of 3 and a median DISTURBED score of 1, indicating very satisfactory visualization quality.

### Cost-effectiveness of ICG fluorescence imaging

Despite the higher direct costs associated with ICG fluorescence imaging—primarily due to the price of the contrast agent (e.g., in the Czech Republic, one 25 mg ampoule of Verdye® costs approximately 160 EUR)—its use may ultimately reduce overall healthcare expenditures. This can be achieved through shorter operative times, reduced rates of bile duct injury, and fewer postoperative complications. A meta-analysis by Thang et al. (2024) [33] evaluated 14 studies involving 3,576 patients and demonstrated that the use of ICG was associated with significantly lower postoperative complication rates (4.78% vs 7.25%; RR 0.71; 95% CI 0.54–0.95; *p* = 0.02), including reductions in bile leakage (0.43% vs 2.02%; RR 0.27; *p* = 0.002) and need for bile duct drainage (24.8% vs 31.8%; RR 0.64; *p* = 0.01). Similarly, a cost-effectiveness model by Reeves et al. (2022) [34] predicted that routine use of fluorescence cholangiography reduces lifetime costs by USD 1,235 per patient and improves outcomes by 0.09 quality-adjusted life-years, largely due to shorter operative times (by over 21 min) and lower conversion rates to open surgery (1.62% vs 6.70%).

### Limitation

This study has certain limitations that should be acknowledged. The sample size was relatively small and reflects the initial clinical experience with ICG-guided laparoscopic cholecystectomy in pediatric patients. Nevertheless, the outcomes were uniformly favorable, and no ICG-related or surgical complications were observed. In addition, although the use of a Likert scale provided valuable subjective insights into the quality of biliary visualization, quantitative fluorescence measurements were not performed. Future prospective studies should consider integrating objective fluorescence metrics and exploring potential correlations with intraoperative decision-making and clinical outcomes.

## Conclusion

Short-interval intravenous administration of indocyanine green is a safe, well-tolerated, and effective adjunct to pediatric laparoscopic cholecystectomy. When administered 3–6 h preoperatively, it enables reliable biliary visualization, enhances anatomical orientation, and supports safer dissection—an advantage that is particularly valuable in pediatric patients, where the overall case volume of cholecystectomy is lower compared with adult practice. This approach is well suited to modern perioperative workflows, including same-day surgery, and may facilitate broader adoption of fluorescence-guided techniques in pediatric practice.

## Supplementary Information

Below is the link to the electronic supplementary material.

Supplementary file1 (PDF 50 KB)

Supplementary file2 (JPG 223 KB)

Supplementary file3 (JPG 205 KB)

Supplementary file4 (JPG 291 KB)

## Data Availability

The datasets generated and analyzed during the current study are available from the corresponding author upon reasonable request.
