# Supplementary material for: Short-interval intravenous indocyanine green administration in pediatric laparoscopic cholecystectomy: a prospective evaluation of visualization and safety
Source: Pediatr Surg Int. 2025 Aug 26;41(1):269. doi: 10.1007/s00383-025-06172-x (PMC12380915; doi:10.1007/s00383-025-06172-x)
Supplement: Supplementary file 1 — Supplementary file1 (PDF 50 KB) [file 383_2025_6172_MOESM1_ESM.pdf]

Supplementary Information - fig. 1
Intraoperative near-infrared fluorescence imaging using indocyanine green (Verdye®). Clear fluorescence of the cystic duct and the common bile duct is visible before peritoneal dissection. ICG was administered at a dose of 0.36 mg/kg, 225 minutes prior to surgery.

Supplementary Information - fig. 2
The different overlay mode. Clear fluorescence of the cystic duct and the common bile duct is visible prior to peritoneal dissection.

Supplementary Information - fig. 3
The cystic duct is clearly visualized (bright green), while the surrounding vessels do not fluoresce. An atypical course of the right hepatic artery is seen running above the cystic duct, with a short cystic artery originating just superior to the duct.
