# Supplementary figures and images for: Short-interval intravenous indocyanine green administration in pediatric laparoscopic cholecystectomy: a prospective evaluation of visualization and safety
Source: Pediatr Surg Int. 2025 Aug 26;41(1):269. doi: 10.1007/s00383-025-06172-x (PMC12380915; doi:10.1007/s00383-025-06172-x)

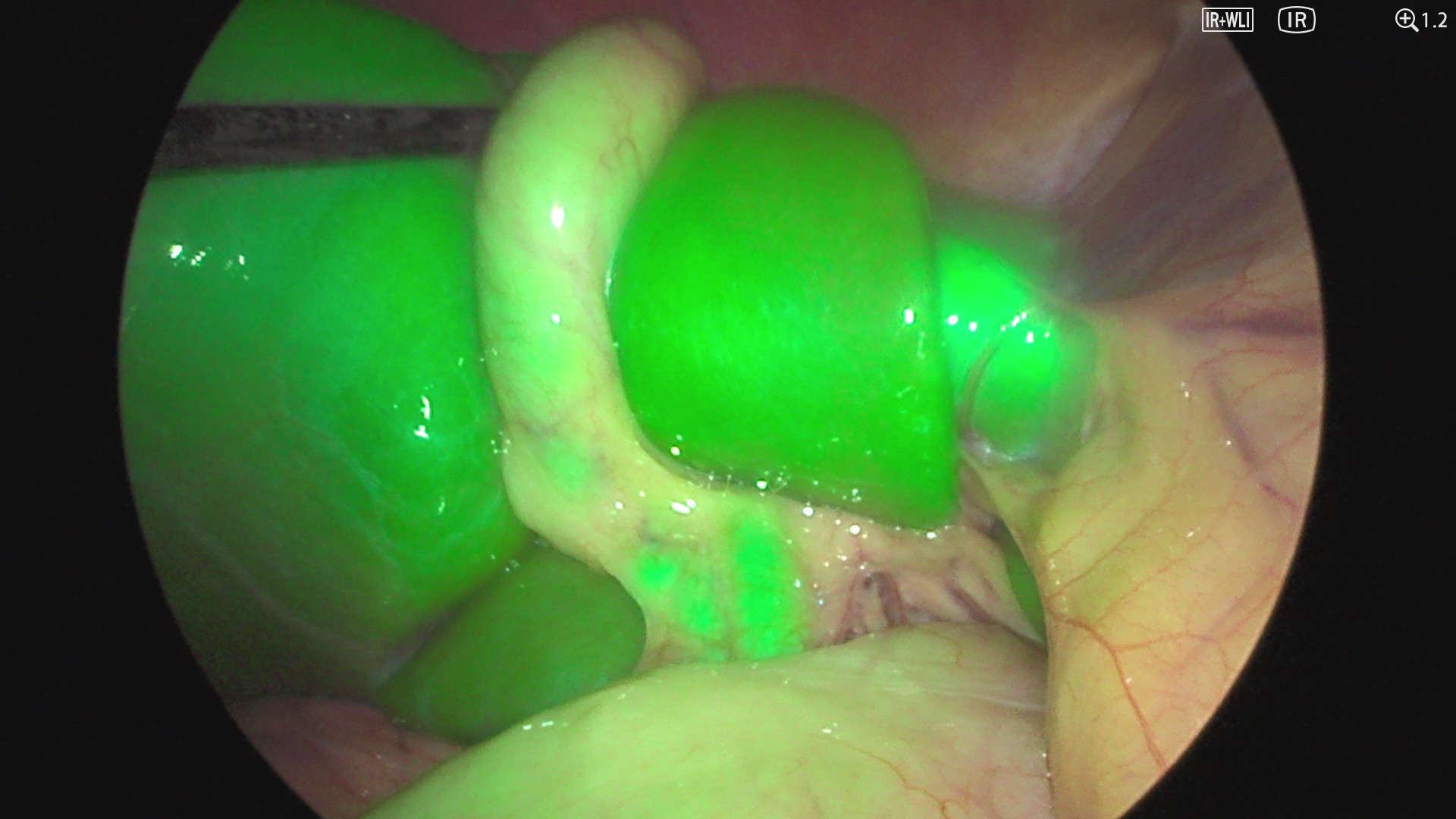

Supplement: Supplementary file 2 — Supplementary file2 (JPG 223 KB) [file 383_2025_6172_MOESM2_ESM.jpg]

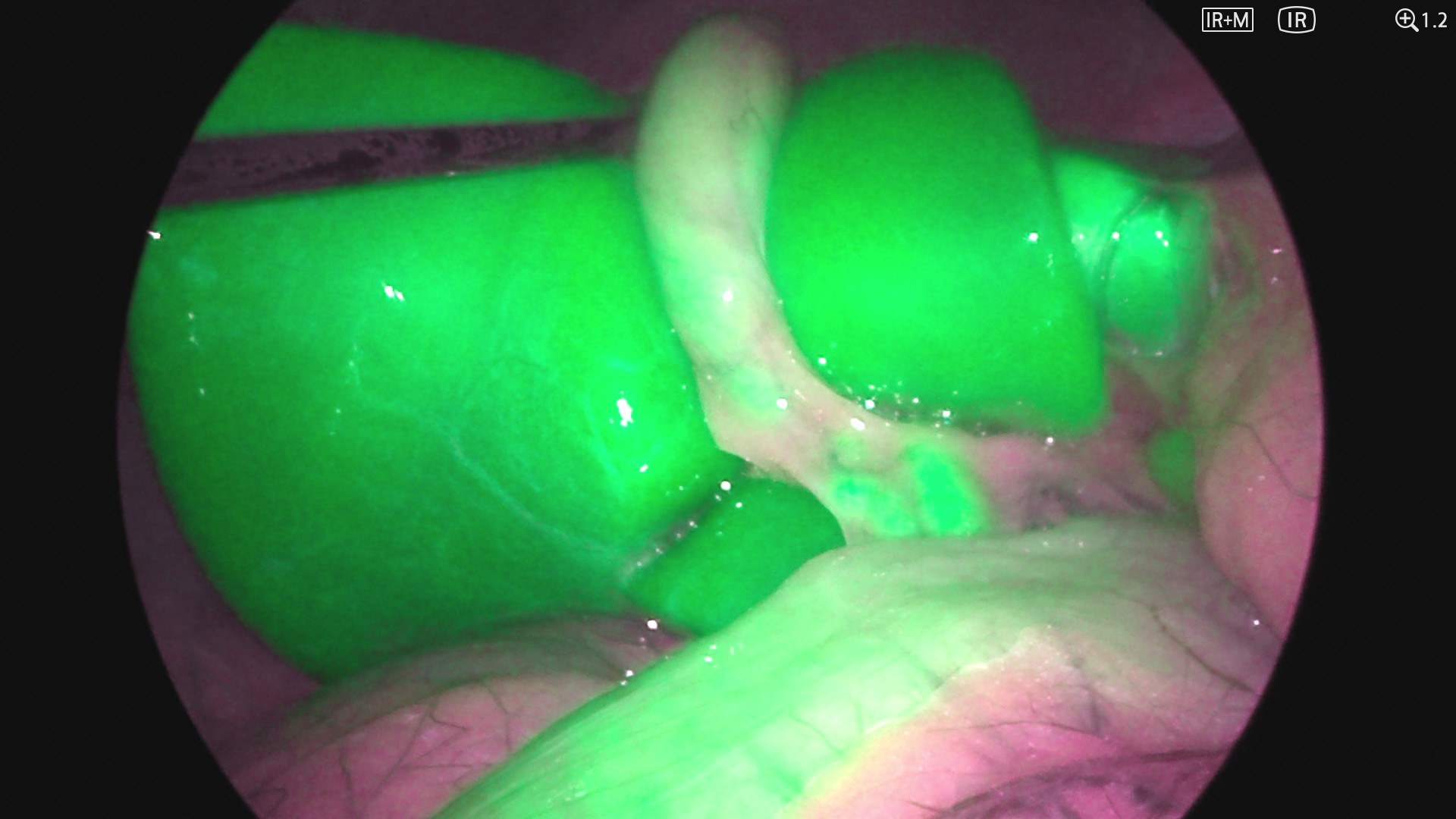

Supplement: Supplementary file 3 — Supplementary file3 (JPG 205 KB) [file 383_2025_6172_MOESM3_ESM.jpg]

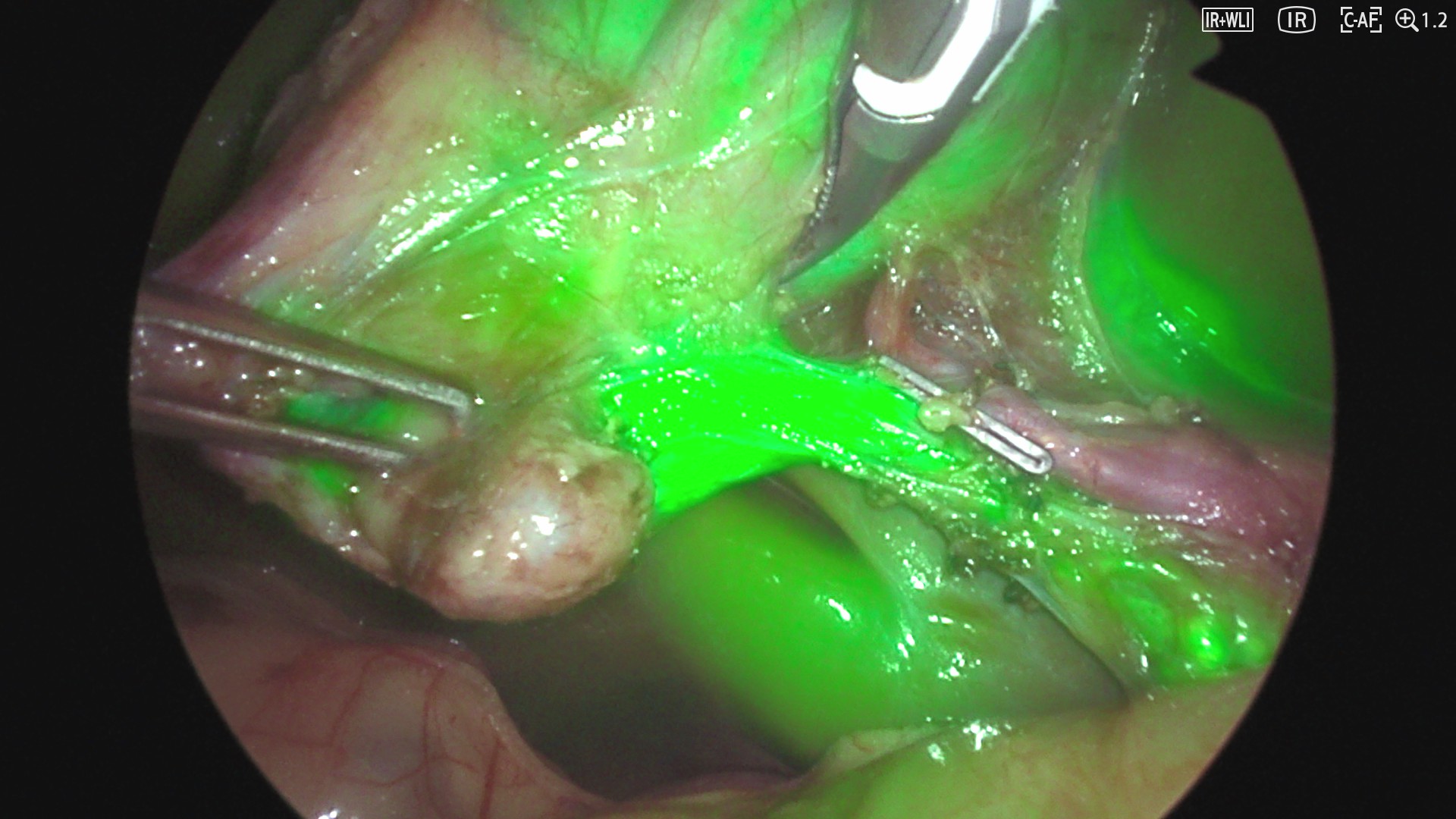

Supplement: Supplementary file 4 — Supplementary file4 (JPG 291 KB) [file 383_2025_6172_MOESM4_ESM.jpg]
